# “…it's so funny to just throw off the blind girl” subjective experiences of barriers in physical education with visually impaired students—an emancipatory bad practice approach

**DOI:** 10.3389/fspor.2025.1515458

**Published:** 2025-03-04

**Authors:** Martin Giese, Michelle Grenier

**Affiliations:** ^1^ Department of Education, Sport Pedagogy, Philipps-Universität Marburg, Marburg, Germany; ^2^ Department of Kinesiology, University of New Hampshire, Durham, NH, United States

**Keywords:** barriers, physical education, ableism, visual impairment, bad practice approach

## Abstract

The objective of this study is to examine the subjective barriers experienced by blind and visually impaired students in general physical education (PE) using qualitative research methods. A total of 10 students, comprising six females and four males, between the ages of 17 and 19 (with an average age of 18.5 years) were interviewed. The students had been enrolled in mainstream schools at the International Standard Classification of Education (ISCED) level 2 and had elected to transfer to a boarding school for the visually impaired at the transition to ISCED level 3. In order to gain insight into the reasons behind the decisions to leave general education schooling and attend a boarding school, an emancipatory bad practice approach was employed. The findings indicate that physical education (PE) is a particularly challenging subject area. The assumption that general education practices and placements yield positive outcomes is contradicted by the interviews, which predominantly document negative experiences. In light of these findings, it is imperative that we examine the reasons for exclusion as experienced by marginalized groups in physical education.

## 1 Introduction

The ratification of the UN Convention on the Rights of Persons with Disabilities (CRPD), enacted in May 2008, obligated educational systems to play a crucial role in overcoming the exclusion of marginalized and discriminated-against societal groups. In this context, the subjective experiences of disabled students regarding barriers to participation in mainstream schooling are of particular interest. In this brief research report, we narrow the focus to the subjectively perceived barriers to participation in physical education (PE) among blind and visually impaired students (BVIS), where students with and without disabilities are enrolled together. The objective is to identify subjective barriers within the school system that explicitly pertain to physical education (PE). The term inclusion is based on an intersubjective conceptualization (1, 2), which models inclusion as *belonging*, *acceptance* and *value* from the perspective of the marginalized persons. This understanding of inclusion is intended to enable an empirical approach to inclusive experiences in order to examine the subjective inclusiveness from the perspective of disabled persons (3).

This exploratory study employs an emancipatory bad practice approach predicated on the assumption that an exclusive focus on best-practice settings is inadequate for developing a comprehensive understanding of the inclusive experience. To gain further insight into the barriers BVIS encounter, and what we consider a gap in the research in general school settings, it is essential to conduct interviews with individuals who may have experienced significant barriers and negative experiences. Interviews were conducted with students who, after nine years enrolled in a general education school, elected to attend a special needs residential school, thereby leaving their parents and the social environment to do so. In light of the significant nature of this decision, this study aims to elucidate the underlying reasons for the students' decision. To achieve this emancipatory goal, it is necessary to amplify the voices of the BVIS, whose school school-careers are characterized by discontinuities. Hence, the exploratory study addresses BVIS reasons for continuing their education in favor of segregated education. This transition period appears to be of particular significance, as research findings indicate an elevated risk of exclusion from the school system, particularly at the transition to ISCED 3 (4, p. 179).^1^

The empirical basis for teaching students with and without disabilities in PE is wide-ranging (5). Previous research is restricted to the perspectives of parents (6), peers without disabilities (7) or of teachers of students in physical education (8). The results demonstrate positive outcomes of inclusive sports lessons (9) and show that these groups tend to have a positive attitude towards inclusive PE (10). It should be noted however, that the perspective of disabled students receives little attention and unquestionable that research from the disabled student's point of view has its own relevance in order to understand whether inclusive practices are considered beneficial or not (11). Accordingly, studies that explicitly look at disabled students' perspective draw a more differentiated picture (12). A considerable proportion of disabled students report unfavorable experiences. As a result, disabled students often strive for recognition as individuals with disabilities, rather than as individuals with unique, undesired, or imperfect bodies (13, 14). These experiences contribute to bullying, social isolation, and other forms of discrimination from teachers and peers. The outcome of these negative experiences can impact a student's attitude regarding their performative skills in sports and physical education leading to a self-selected withdrawal from future sports activities (15).

## 2 Method

The data presented here were derived from a research project whose aim was to identify barriers related to educational decisions that BVIS perceives in general education settings. The project was not focused on a particular school subject; rather, it aimed to identify general barriers in a school for BVIS. However, given that in nearly all interviews, physical education (PE) was explicitly identified as a particularly problematic area, this brief research report focuses specifically on the subjective constructions of sport-related participation in PE. Therefore, the original data, which has already been published (16), were reanalyzed with a particular emphasis on identifying the factors that hinder participation in physical education.

In the light of the preliminary considerations outlined, two research questions informed the re-analysis:

• How do BVIS reflect on their sports lessons with peers without disabilities?
• What barriers to participation are visible in these constructions?

### 2.1 Sampling

The data were collected at a state-recognized special school for visual impaired students. The school follows the aim of general university entrance qualification. The school has a boarding school attended by most of the students. A total of nine pupils between ages 17 and 19 (average: 18.1 years) of the upper secondary school (Grade 12) took part in the research (Table 1). According to the social law classification in Germany, they were visually impaired and had no additional disabilities. All interviewees have agreed to participate in the study on the basis of detailed study information. IRB approval with pseudonyms were used when reporting the data. According to the bad-practice approach, only students enrolled in general educaiton schools close to their place of residence during the entire ISCED 2 who explicitly opted for switching to a special school and the associated boarding school in the transition to ISCED 3 participated in the research. The intervieews were asked to explicitly talk about their experiences in general education.

**Table 1:** Characteristics of the respondents (on the date of the interview).

Name	Age	Gender	Degree of VI
Tim	19	Male	Blind
Kian	19	Male	Visually impaired
Susanne	19	Female	Blind
Anna	17	Female	Visually impaired
Larissa	17	Female	Visually impaired
Katharina	17	Female	Visually impaired
Julius	18	Male	Visually impaired
Franziska	18	Female	Visually impaired
Sarah	19	Female	Visually impaired

### 2.2 Data collection and analysis

Based on Rabenstein and Gerlach (17), educational decisions are seen as optimisation processes that last through one's entire school career. For the reconstruction of these processes, episodic interviews were used (18, p. 278), which aim at “changes from the point of view of respondents, but without placing a clear and exclusive focus on biographical processes”. The episodic interview targets situational narratives of interviewees (18). All interviews were digitally recorded and transcribed. Data analysis was performed with the software MAXQDA 2024 which structures qualitative content analysis into deductive-inductive category formation recommended for the evaluation of episodic interviews (19). In the upper category *general statements on PE* barriers and resources were recorded, which can be seen in the subjective constructions of the subjects. The other four upper categories *body and performance*, *didactics*, *special educational services*, and *social relationships* were formed deductively based on the state of research as well as the everyday knowledge of the researchers. The differentiation of the category system with the subcategories was carried out inductively. Two independent encoders were involved in the coding process, with parts of the material being independently coded twice at the beginning of the process. Consensual encoding was selected as a procedural method to ensure consistent coding (20).

## 3 Results

To facilitate a structured discussion of the results and to focus on the context of PE, three thematic foci have been selected and used in the results section. Given the nature of the brief research report, the results are presented in an exemplary manner. The thematic foci are: (a) general statements on general education, (b) social relationships, and (c) body, performance, and didactic understanding.

### 3.1 General statements on general physical education

In general, most pupils reported that the perceived barriers in general education increased over the course of their time at school.

Yes, everything used to be normal in elementary school. Everything was still fine then. I got on really well with everyone, I got on well with everyone. And it was the same the other way around. And then the tide turned in secondary school. (Tim #81)

While general education schooling was described as unproblematic in Years 5 and 6, it is worth noting that the perceived barriers gradually increased from Year 7, with the greatest challenges emerging at the end of ISCED 2 (Year 10). Support from parents or friends is seen as the most important resource in overcoming barriers. As reported by Kian, for example, who described an intereaction with a friend: “Come on, we’ll train a bit after school too, so you won’t be so disadvantaged in PE lessons” (Kian #41). The emotional stress experiences and correlated with physical stress, including in sports lessons:

So the main reason was always that I, I always did everything myself, so after school. At some point, it got to the point where I was doing fitness in PE, so I also did fitness privately and so on, that at some point I really broke down and I was completely exhausted, especially because of the visual impairment, dealing with the whole situation and then all the stress at school. (Kian #135)

Seven out of nine interviewees indicated a correlation between the degree of visual impairment and the perceived level of personal suffering. In particular, the pressure of distress increased when there was a deterioration in vision.

And the fact that my vision got worse made it even worse. And above all, when I was playing ball, for example, it was very often the case that because my field of vision is also quite small, I was basically constantly flying over [..] these little hats or I couldn't see the basket. (Susanne #59)

Physical education appeared to be a catalyst in which problems were centered on the body and was repeatedly cited as a key barrier at school:

So, I found the main barriers were the structure of the lessons. And the sports lessons. And reading aloud. Well, those were three things that I really noticed. At some point, I gradually became less able to recognize balls and that naturally had an impact on my classmates. (Anna #23)

The experiences cause Susanne (#27) to have a chronic fear of physical education.

### 3.2 Social relationships

In addition to the general statements, social interaction with teachers, peers and special education services played a central role in the interviews. Forms of perceived otherness were described as particularly negative in physical education. The feeling of otherness was triggered, for example, by the teacher assigning special roles or by the explicit exclusion from group activities.

And then my personal assistant somehow threw me a ball on the side, a big one or something. That was just kind of stupid. (Larissa #47)

Katharina reported that she perceived her teacher's actions to be characterized by intolerance, indifference and active exclusion when she “mostly never took part in PE lessons. Because the teacher said, yes, you can’t see anyway, then you’d better not join in” (Katharina #25). Franziska (#124) reported a lack of recognition of her visual impairment by her PE teacher, who “didn’t quite realize that I have a visual impairment”. Tim was also not allowed to take part in PE lessons because “the time was used for subjects in which I had problems” (Tim #73). In addition, the special educational support systems also proved to be barriers to participation. The expertise of the special education service teams was minimal in physical education. As Julius (#59) asked rhetorically: “Yes, but what can they say about physical education?” Moreso, peer relationships in particular proved to be massive barriers. In almost all interviews, rejection by peers and active acts of bullying were reported. For example Sarah reported (#123) “I was often thrown off in sports, simply because it's so funny to just throw off the blind girl who can’t defend herself anyway, who didn’t have any friends anymore.” In several cases, the teachers knew about the bullying and did not intervene or were perceived as reinforcing the situation:

A blind man with a walking stick can see that. And of course, in front of my classmates. They immediately realized: Aha, now we have a teacher who sees things the same way we do! Can we somehow get together with him or, yes, cooperate somehow? (Tim #97)

Overall, it can be seen that the social inclusion of visually impaired students degraded as the grade level increased. Julius (#55) laconically summarized that “the not so great understanding of my classmates [..] „just wasn’t so practical” and Anna (#61) noted with resignation that she “was always the one who was like: Oh, now we have to have them on the team”. Katharina also justified her own withdrawal from PE lessons with the perceived rejection by her non-visually impaired peers, “that's why I was like..they just didn’t fancy me. Yes, I was a burden” (Katharina #41).

### 3.3 Body, performance, and didactics

The third set of issues proven to be problematic was the focus on traditional ball games and individual sports: “Yes, then of course the classic PE lessons” (Julius #45), which correlate with ableist ideas of performance and the body and are strongly oriented towards competitive sports (21).

… because we played ball games very often. And I felt like I had a target on my face. And either I didn't see where I was actually throwing and didn't see my teammates or I didn't see when a ball was flying towards me, things like that. (Susanne #27)

It is noteworthy that Katharina described the impression that teachers tried to compensate for their situation by giving positive grades. “Like in PE, because of badminton and everything. There's just a three, because a three is a three. You can't complain about that.” (Katharina #95).

I always found it negative that many people didn't want to learn to understand it. We had PE where at some point it was clear, okay, I can't catch the ball. Someone could have said, okay, then I'll go into the goal or something. Or at badminton. I told my teacher that I wasn't able playing. It would have been great if they had been a bit more understanding. (Anna #61)

Regarding efforts towards an inclusive sports pedagogy, interviewee excerpts show that it seemed necessary to further analyze the complex relationships between immanent subject-didactic convictions of teachers, basic educational-theoretical assumptions in sports pedagogy and subjective constructions of (marginalized) learners in order to identify exclusive potentials as precisely as possible.

## 4 Discussion

In alignment with the conclusions presented by Haegele and Kirk (22), a synopsis of the results indicates that physical education (PE) is perceived as a highly challenging subject. The physical, bodily, performance-oriented, and content-related barriers to participation in PE are evident. Forms of perceived otherness are described as being particularly negative (14). Such exclusionary tendencies are reinforced by the fact that the socialization of teachers in the context of sports, which has often taken place in club and competitive sports (21), obviously hinders the recognition of different bodies. In contrast with the assumption that effectively implemented inclusive practices typically result in positive experiences for disabled students in general physical education (23), negative experiences were pervasive in the interviews. Consequently, it is imperative to prioritize a more rigorous examination of the exclusionary potential of physical education, particularly from the perspective of marginalized groups (24). With regard to visually impaired students, the degree of visual impairment and phases of visual deterioration serve as additional catalysts for exclusionary processes.

It also appears fundamental that the perception of barriers increases over the years of the attending school, and at the same time, the specific needs of the respondents received less and less attention (25). As Jessup et al. (26) also show, engagement with pupils can function as an important resource but can also be ruinous. As a result of increasing overload and personal devaluation by peers and teachers, psychosocial problems and eroding self-esteem arise. If, in addition, special education services fail or have a counterproductive effect, a multicausal system of failure occurs (16). As Jessup et al. (27) also report, in almost all cases, rejection by peers as well as active bullying behavior was reported (28). Paradoxically, it should be noted that these experiences of exclusion are also said to have an ambivalent learning potential because, as Anna (#85) puts it, they help to “get through real life somehow.”

The study results confirm theoretical and empirical findings that the needs of visually impaired students are simply omitted and that ableist body and performance norms have a potential for exclusion (29, 30). In a constructivist turn, addressing and negotiating these norms in physical education in more individualized ways would be an important step in promoting the inclusivity of physical education.

In terms of limitations, it should be mentioned that the bad practice approach was used to interview only subjects who had left inclusive schooling and had deliberately switched to a special institution with residential accommodation. Due to this pre-selection, there are no voices of subjects who consider their inclusive schooling a success, which also suggests that the results are particularly concentrated here. This results in further research desiderata. For example, students with additional disabilities and/or other marginalized groups should also be interviewed in the context of intersectionality to clarify whether something like collective experiences of exclusion can be reconstructed beyond isolated observations of individual forms of disability.

## Data Availability

The data analyzed in this study is subject to the following licenses/restrictions: It will be deleated in three years and it is strictely restricted to the authors. Requests to access these datasets should be directed to Martin Giese.

## Footnotes

^1^Developed by UNESCO in the mid-1970s, ISCED is the reference international classification for organizing educational programs and related qualifications by level and field.
